# A novel method of simulated-use surface disinfection efficacy testing as Phase 3 Step 1 approach

**DOI:** 10.1016/j.infpip.2026.100511

**Published:** 2026-01-20

**Authors:** A. Ulatowski, B. Knobling, D.C. Mogrovejo, J.K. Knobloch, F.H.H. Brill

**Affiliations:** a Department of Bacteriology, Dr. Brill + Partner GmbH Institute for Hygiene and Microbiology, Hamburg, Germany; b Institute for Medical Microbiology, Virology and Hygiene, Department Infection Prevention and Control, University Medical Center Hamburg-Eppendorf, Hamburg, Germany

**Keywords:** Surface disinfection, Phase 3 testing, Simulated-use-testing, Wipes, Clinical isolates, Touch transfer

## Abstract

**Background:**

Standard laboratory tests for surface disinfectants often fail to reflect real-life clinical conditions, potentially overestimating efficacy. Simulated-use testing that incorporates clinical strains, realistic contamination and user application may provide a more accurate reflection of in-use performance in healthcare settings.

**Aim:**

The aim of this study was to develop and validate a standardized, reproducible Phase 3 Step 1 simulated-use surface disinfection test that incorporates clinically relevant organisms, hospital-representative surfaces, and realistic application methods.

**Methods:**

Based on EN 16615:2015, the test method was modified to reflect hospital conditions more closely. Clinically isolated outbreak strains of *Staphylococcus aureus*, *Enterococcus faecium* and *Acinetobacter baumannii* were used. Contamination was applied via a touch-transfer method. Surface materials included hospital-relevant substrates, and disinfectant wipes were applied by trained volunteers to simulate routine cleaning practices.

**Findings:**

The touch-transfer contamination method was reproducible, and no significant differences were observed in drying or water controls across different surfaces. Wiping speed and contact pressure did not correlate with efficacy. However, microbial recovery varied across test runs and participants. The test method presented here allows for efficacy testing of commercial disinfectants.

**Conclusion:**

A Phase 3 Step 1 simulated-use test was established, which incorporates micro-organisms isolated from the application area, surfaces representative of the application area, and where the product is applied by trained participants. This internally validated method better represents clinical disinfection practices compared with current standardized tests and may support improved assessment of surface disinfectant efficacy under conditions approximating real-world hospital use.

## Introduction

Pathogens causing healthcare-associated infections (HAIs) can survive on a wide range of surfaces in healthcare environments, despite routine cleaning and disinfection following recommended protocols [1]. Discrepancies between test conditions, the complexity of hospital environments and insufficient protocol compliance contribute to pathogen persistence and increased HAI risk, particularly in rooms previously occupied by infected or colonized patients [1,2]. Effective removal of microbial contamination from frequently touched surfaces relies on mechanical removal of dirt and the proper use of antimicrobial products [3], with correct formulation, manual distribution and adherence to contact times being crucial [4].

In-vitro studies and standardized procedures such as Phase 1 tests (quantitative suspension test for products under development), and Phase 2 tests (Step 1, quantitative suspension tests, or Step 2, quantitative carrier tests), allow efficacy testing by defining exposure times and active compound concentrations to achieve required microbial reductions [5,6]. However, multiple factors influence disinfectant performance [7], and laboratory-developed protocols often fail to reflect real-use conditions [5]. Moreover, efficacy is reduced when comparisons between practice-like conditions and laboratory settings are carried out [8], even when low microbial loads are present [9]. Laboratory efficacy tests typically use defined standard organisms, which may not represent the diversity of clinical isolates encountered in healthcare settings [10]. Testing against naturally occurring pathogens provides more meaningful results [[11], [12], [13]].

Phase 3 testing addresses these limitations by assessing disinfectant performance under realistic conditions, either through simulated-use or field tests. Field tests evaluate efficacy against natural microflora without artificial contamination [14], but results depend on variable microbial loads and environmental factors, requiring multiple measurements for reliable assessment [14].

Since the current European standard EN 14885:2023 does not differentiate between field studies and simulated-use tests, the aim of this study was to develop a realistic, reproducible, laboratory-based Phase 3 Step 1 (simulated-use) surface disinfection efficacy test [6] incorporating realistic test surface materials from hospital environments, realistic clinical test organisms and a realistic application to test surfaces and realistic disinfectant application by trained volunteers.

## Methods

The simulated-use method developed in this study was adapted from the EN 16615:2015 methodology [15] and follows the guidance of the draft norm EN 14885:2025 [6] regarding Phase 3 Step 1 test.

The European standard EN 16615:2015, also known as the 4-field test, quantitatively assesses surface disinfectant efficacy using mechanical wiping. Four 5 × 5-cm fields are marked in a row on a 20 × 50-cm polyvinyl chloride (PVC) test surface. Field 1 is contaminated with a microbial suspension, and the test surface is stored at 20 °C for drying (relative humidity between 40–60%) until visibly dry. A wipe, attached to a standardized weight, is used to wipe from field 1 to 4 and back. After the required contact time, each field is sampled using a swab soaked in neutralizer, followed by a dry swab. Swabs are vortexed in neutralizer solution, from which test organisms are recovered by plating and incubation.

### Method development

**Figure 1:**
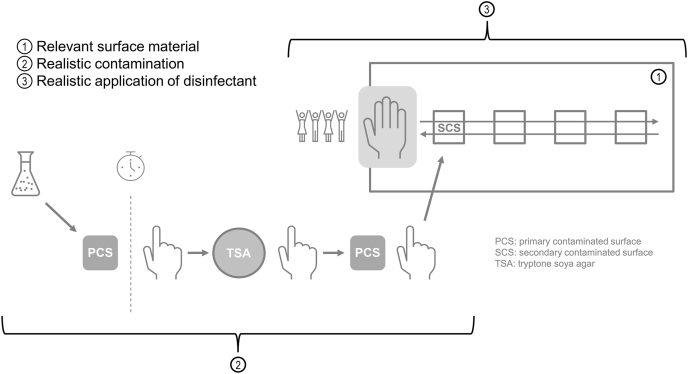
Overview of the simulated-use test, a methodology based on EN 16615:2015 with modifications for (1) the surface material, (2) contamination method of the test surface (touch transfer) and (3) application of the disinfectant. The touch-transfer contamination is performed by inoculating a primary contaminated surface (PCS) (1) with the test organism and letting it dry. The test organism is then transferred with a gloved finger from the PCS to the SCS (field one of the test surface) (2). Once the SCS is dry, a test person wipes once from test field 1 to test field 4 and back (3). After the contact time, the test organisms are recovered, plated and incubated according to EN 16615:2015. SCS, secondary contaminated surface. Modified from prEN 14885:2025 [6].

In this study, the modifications to the EN 16615:2015 were made to 1) the surface material, 2) the contamination of the test surface and 3) the application of the disinfectant. These modifications are presented in Figure 1 and are described as follows:

#### Surface material

This study provides three durable, non-dangerous, cost-effective alternatives to PVC with polyurethane (PUR) surface coating used as test surface in EN 16615:2015. These materials also represent frequently touched surfaces in the hospital environment: a) acrylonitrile butadiene styrene (ABS) copolymer, (S-Polytec GmbH, Germany), commonly used, for example, in medical device storage, medical instrument handles and light switches; b) melamine, solid plastic panels coated with melamine resin (BSO Montagetechnik GmbH, Germany), used mainly in furniture such as bedside tables, working tables, bed frames; and c) stainless steel (MAB GmbH, Germany), widely used in hospital environments as the main, if not only, material in working and operating tables, door handles, bed rails, etc. The test surfaces were precleaned with 70% isopropanol (Carl Roth GmbH, Germany) and used only once.

#### Contamination of the test surfaces

Test surfaces were contaminated with either i) a clinical isolate – methicillin-resistant *Staphylococcus aureus* (MRSA), vancomycin-resistant *Enterococcus faecium* (VRE) or *Acinetobacter baumannii*, ii) the reference strain *Staphylococcus aureus* ATCC 6538 or iii) a mixed inoculum consisting of MRSA and VRE.

The clinical isolates were selected from a well-characterized multi-drug-resistant strain collection of the University Hospital Hamburg-Eppendorf, UKE (Hamburg, Germany). The reference strain *S. aureus* ATCC 6538 was obtained from the German Collection of Micro-organisms and Cell Cultures (DSMZ GmbH, Germany). After receiving the isolates, they were maintained according to the EN 12353:2021-11 [16]. Further information on all isolates described in this study is presented in Supplementary Materials (Part 1. Information about isolates).

Test suspensions were prepared according to EN 16615:2015, using a validated initial cell count of 10^9^ colony-forming units per millilitre (cfu/mL) without organic load. For mixed inoculum tests, individual suspensions were prepared and combined in equal volumes.

Based on the touch transfer assay [17,18], a first surface (5 × 5-cm ceramic tile, white matt glaze)—the primary contaminated surface (PCS)—was inoculated with 20 μL of the test suspension, spread evenly on the PCS with a spatula and stored at 20 °C until the PCS was visibly dry. The touch-transfer procedure involved pressing a gloved forefinger—a disinfected nitrile glove (SLN 420, Heliomed Handelsges.m.b.H, Austria) under a sterile cotton glove (Ansell StringknitsTM 76-100, Ansell Healthcare Europe NV, Belgium)—first on a tryptic soy agar (TSA) plate, then on the PCS, and finally on the secondary contaminated surface (SCS) for 10 s each and exerting approximately 100-g pressure (Figure 1). Moisture from the TSA plate optimized organic load transfer.

The test surfaces were either the first test field of the SCS (when the surface was wiped with the product or water) or the drying control (Dct), performed according to EN 16615:2015 Three test surfaces for each material were used. Once visibly dry, test surfaces were wiped as described as follows.

#### Application procedure for the disinfectant

After contaminating the test surface, disinfectant was applied (Figure 1), by 6 test persons. Three separate test surfaces were contaminated, and participants were instructed to wipe the surface directly with the wipe in their (gloved) hand from test field 1 to 4 and back without turning after field 4. No further instructions about wiping speed or the pressure applied to the surface were given.

Multitex® Safe & Clean Wipes DR (ZVG Zellstoff-Vertriebs-GmbH & Co. KG, Germany) were chosen as wiping material. The wipes were soaked for at least 30 min with 23 mL of hard water with polysorbate 80 or one of the two disinfectants described in Table I. Preparation of polysorbate 80 and hard water are described in Supplementary Material (Part 2 Solution preparation). The volume needed to fully soak the wipes, 23 mL, was determined from the manufacturer's instructions and through previous validations of the wipes (data not shown).

**Table I:** Test conditions, disinfectants and neutralizer used

Disinfectant (product type)	Product components^a^	Contact time	Concentration^b^	Neutralizer
A (quaternary ammonium compound + amine)	12.5 g/100 g didecyldimethylammonium chloride + 1.5 g/100 g N-(3- aminopropyl)-N-dodecylpropane-1,3-diamine	30 min	0.005%	TLSH-Nt^c^: 3.0% polysorbate 80; 0.3% saponin; 0.3% lecithin; 0.1% histidine; 0.5% sodium thiosulphate.
B (alcohol + amine)	58.6 g/100 g ethanol 94% + 0.03 g/100 g amine	1 min	50%	

a As detailed by the manufacturer.

b Ineffective concentration according to manufacturer.

c All reagents from Carl Roth (Germany).

Ineffective concentrations of products were used during method development to observe differences in strain behaviour. Concentrations that did not allow survival of test strains allowed no comparisons and were therefore not useful for test development.

In contrast, efficacy tests with effective concentrations of the disinfectant were used for validating the stability over time (>6 months) of the isolates used in the study (Supplementary material, Part 3, Supplementary Figures S1 and S2).

### Method validation

#### Validation of surface and wiping materials

The goal for the validation of the surface as well as the wiping materials is to recover enough test organisms from both the water (N_W_) and drying controls (Dc_t_) to meet the criteria specified in the EN 16615:2015 and to determine that such recovery does not differ significantly between the different materials.

Water and drying controls were performed with *A. baumannii* as an exemplary test organism (drying-resistant Gram-negative species). For the drying controls, one field (5 × 5 cm) of the test surface was contaminated with test suspension via touch transfer as described above and stored for drying at 20 °C. For water controls, field 1 of the test surface was contaminated and dried in the same manner and the wiping was carried out by one person. After an exposure time (or drying time for the drying controls) of 30 min, *A. baumannii* was recovered from the test fields by plating and incubation as described in EN 16615:2015.

The validation limit for the water control was set to 10^2^ cfu to ensure meeting the criteria of >10 cfu on fields 1-4 after the water controls (based on EN 16615:2015). For drying controls, the validation limit was set to >10^4.5^ cfu, to calculate a 4-log reduction on test field 1 by the disinfectant, the requirement for sufficient bactericidal activity in the simulated-use test.

To determine how high the initial cell count must be to meet these validation criteria, water and drying controls were performed on ABS with different initial cell counts from 10^6^ to 10^10^ cfu/mL. After validation, all further controls and simulated-use tests were conducted with the validated initial cell count of 10^9^ cfu/mL.

#### Validation of disinfectant application by several test persons

To assess how variation in wiping technique affects disinfectant efficacy, a simulated-use test was conducted with six test persons, each wiping three ABS test surfaces inoculated with *S. aureus* ATCC 6538, prepared as described previously. Test persons wiped the surfaces using a Multitex® wipe soaked in disinfectant B (Table I) following the procedure described earlier. An ineffective concentration of disinfectant was used so that differences between the test persons, if present, could be observed. Wiping duration was recorded with a stopwatch to measure speed, and the highest contact pressure was recorded using a scale (Smart Weigh ACE200, Better Basics, USA) placed beneath the surface during wiping.

#### Validation of clinical isolates

The three clinical isolates were validated in this study regarding their usability and stability over time by regularly performing drying curves and simulated-use tests with an ineffective and an effective concentration (according to manufacturer) of disinfectant A over a period of approximately six months (Supplementary material, Part 3 Validation over time).

#### Validation of the use of a mixed inoculum

To evaluate the usability of the mixed inoculum, a simulated-use test with a bacterial suspension consisting of MRSA and VRE was performed. The mixed inoculum was prepared from two test suspensions with initial cell counts of 10^9^ cfu/mL and subsequently mixed and applied to three ABS test surfaces in duplicate as described earlier. Once test surfaces were visibly dry, they were wiped with a Multitex® wipe soaked with disinfectant A (0.005%, ineffective concentration). After 30-min exposure, the test organisms were quantified by plating and incubating according to EN 16615:2015. MRSA and VRE were counted separately as they are easy to distinguish based on morphology: MRSA as big, yellowish colonies, whereas VRE colonies are small and white.

### Statistical evaluation

All data were checked for normality using the Shapiro–Wilk test. One-way analyses of variance followed by Tukey’s multiple comparison tests were used for validation of test material by determining if recovery of *A. baumannii* after drying and water controls differed significantly on different surface materials as well as by comparing bacterial recovery after wiping the test surface by several test persons.

Spearman correlations, 95% confidence level, were performed for disinfectant application to evaluate correlation of recovery of *S. aureus* ATCC 6538 in simulated-use tests and different contact pressures and/or wiping speeds during wiping performed by several test persons.

GraphPad Prism version 10.1.2 for Windows [19] was used for all statistical evaluations and figure generation.

## Results

### Validation of surface and wiping materials

**Figure 2:**
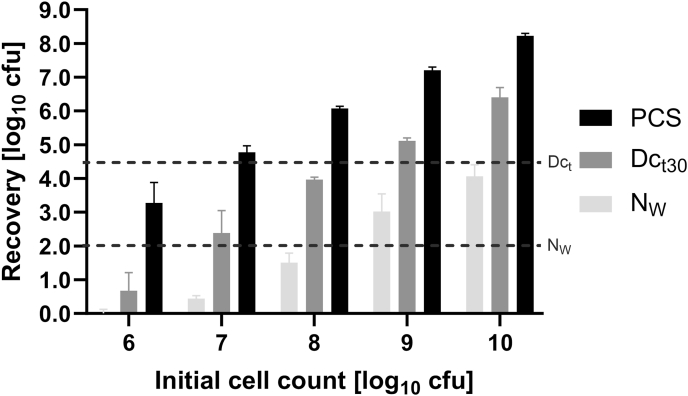
Recovery of *A. baumannii* from the PCS (black bars), from the SCS drying controls (Dc_t30_) (dark grey bars) and the SCS water controls (N_W_) (light grey bars) after 30 min in a simulated-use test on ABS plates with initial cell counts between 10^6^ and 10^10^ cfu/mL (*N* = 4). Only initial cell counts of 10^9^ and 10^10^ cfu/mL yield bacterial recovery within validation limits. ABS, acrylonitrile butadiene styrene; PCS, primary contaminated surface; SCS, secondary contaminated surface.

The recovery of *A. baumannii* from the PCS as well as after a drying and exposure time of 30 min in the drying and water controls increased approximately tenfold with each tenfold increase of the initial cell count. Only with initial cell counts of 10^9^ and 10^10^ cfu/mL, the recoveries of *A. baumannii* in both the drying and water controls were within the validation limits of >10^4.5^ and >10^2^ cfu, respectively (Figure 2).

**Figure 3:**
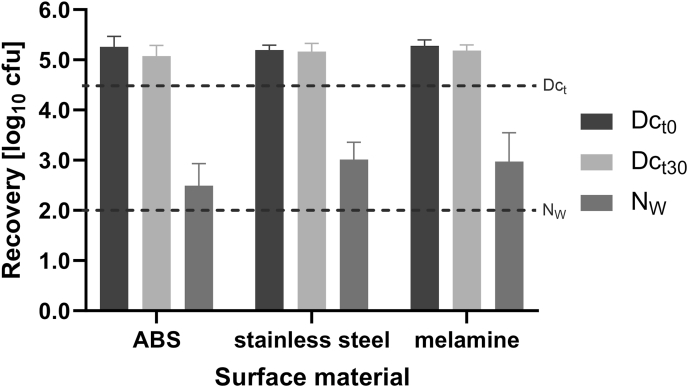
Recovery of *A. baumannii* after drying (Dc_t_) and water controls (N_W_) with 0 (black bars) and 30 min drying time (light grey bars) and 30 min exposure time (dark grey bars) in a simulated-use test on ABS, stainless steel and melamine plates with Multitex*®* wipes (*N* = 6). Dotted lines mark the minimum recovery in the water and drying controls set for the simulated-use test. There were no significant differences between the three surface materials.

As a result, further validation of the surface materials in combination with Multitex® wipes was conducted with an initial cell count of 10^9^ cfu/mL of *A. baumannii*. For that, drying and water controls according to the simulated-use test were performed on these surface materials with drying times of 0 and 30 min and an exposure time of 30 min in the water controls (Figure 3). All drying and water controls were above the limit of 10^4.5^ for the drying and 10^2^ cfu for the water controls, and there were no significant differences between the three surface materials (*P* = 0.5900 (Dc_t0_), *P* = 0.05110 (Dc_t30_) and *P* = 0.1322 (*N*_W_)).

### Validation of disinfectant application by several test persons

**Figure 4:**
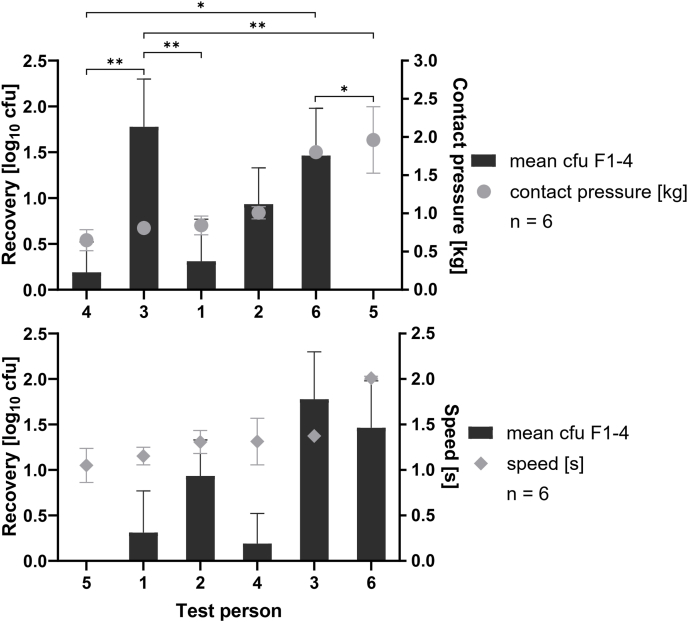
Recovery of *S. aureus* (bars) after a simulated-use test with disinfectant B and Multitex® wipes on ABS plates. The wiping process was performed by six different test persons, in triplicate, and there was no correlation between the recovery of *S. aureus* and the contact pressure (grey circles) or the speed (grey squares) of the wiping process. There were significant differences in the recovery of *S. aureus* among the test persons overall (*N* = 6).

The individual variances in the wiping technique of different people and their influence on the efficacy of surface disinfectants were evaluated in a simulated-use test performed on an ABS test surface contaminated via touch transfer with a test suspension of 10^9^ cfu/mL *S. aureus*. Simultaneous to the wiping process, the contact pressure that each test person applied with the wipe on to the test surface as well as the duration of the wiping process (speed) was recorded (Figure 4).

There was no correlation between the recovery of *S. aureus* and contact pressure (95% confidence interval [CI]: -0.95; 0.89, *P* = 0.7139) or speed of the wiping process (95% CI: -0.6652; 0.9933, *P* = 0.1028) between the test persons. There were significant differences in the recovery of *S. aureus* among the test persons overall (*P* = 0.0011) as well as in the pairwise comparison (*P*-values ranging from 0.0021 to 0.0444 [test persons 3 vs 5 and test persons 4 vs 6, respectively]).

### Validation of clinical isolates and use of a mixed inoculum

The clinical isolates – MRSA, VRE, and *Acinetobacter baumannii*—were stable over time as demonstrated by their consistent performance in efficacy tests (performed according to the simulated-use test developed in this study) and drying curves carried out regularly during a period of greater than six months (results shown for isolates after 1.5 and 7 months—Supplementary Material, Part 3, Figures S1 and S2).

**Figure 5:**
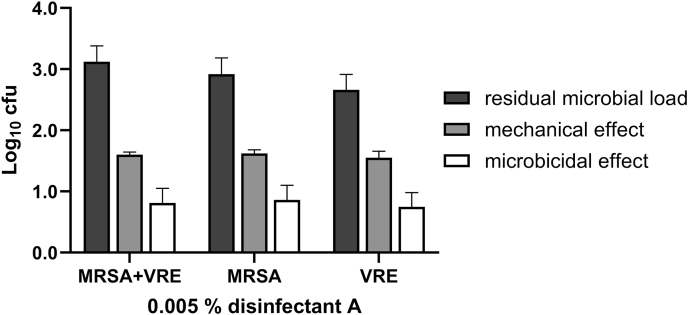
Residual microbial load (black) of MRSA and VRE isolates and microbicidal (white) and mechanical effect (grey) of disinfectant A against a mixed inoculum of MRSA and VRE in a simulated-use test on ABS plates (*N* = 6). The two isolates were counted and shown in the graph separately and together. The microbicidal and mechanical effects were calculated based on the recovery in the drying control Dct30. For VRE, the drying control Dct30, the residual microbial load on test fields 1-4 as well as the initial cell count were lower than for MRSA. The mechanical and the microbicidal effects were also slightly lower for VRE (1.55 ± 0.11 and 0.75 ± 0.23 log10) than for MRSA (1.62 ± 0.06 and 0.86 ± 0.24 log10). MRSA, methicillin-resistant *Staphylococcus aureus*; VRE, vancomycin-resistant *Enterococcus faecium*.

Regarding the use of a mixed inoculum consisting of MRSA and VRE, Figure 5 shows the residual microbial loads on fields 1-4 of the isolates (separate and combined) as well as the mechanical and microbicidal effect of the water control N_W_ and disinfectant A calculated from the drying control Dct_30_.

For VRE, the drying control Dct_30_, the residual microbial load on test fields 1-4 as well as the initial cell count were lower than for MRSA. The mechanical and the microbicidal effects were also slightly lower for VRE (Figure 5).

The aforementioned criteria for validating surface and wiping materials as well as the isolates themselves were met with the recovery of VRE and MRSA being all above of 10^4.5^ and 10^2^ cfu after the drying and the water controls, respectively. Therefore, the isolates VRE and MRSA can be used in a simulated-use test as single test organisms or in a mixed inoculum.

## Discussion

Standardized efficacy tests provide reproducible laboratory conditions to evaluate biocidal products [14]. However, current Phase 2 Step 2 methods (e.g. EN 16615:2015) may not reliably predict performance under real-world conditions [5]. Recognizing this, the EN 14885:2022 and EN 14855:2023 point out that Phase 3 tests—field or simulated-use studies—are valuable tools to assess disinfectant efficacy under realistic conditions [6,14].

This study established an internally validated Phase 3 Step 1 simulated-use test that incorporates healthcare-relevant surfaces, clinical isolates, practice-like contamination via touch transfer and product application by multiple test persons, all relevant factors that define a Phase 3 Step 1 test [6]. Touch-transfer contamination was observed to be reproducible as reported previously [20], and adjusting the PCS inoculum allowed precise control of SCS contamination (aligning with Phase 2 requirements of EN 16615:2015 for instance). Drying and water controls across surface types showed no significant differences, supporting the method's applicability to diverse healthcare surfaces.

During product application, no correlation was found between wiping speed or pressure and organism recovery. However, differences among test persons in recovery outcomes highlight the importance of involving multiple users in testing. The differences observed, although statistically significant, were practically irrelevant as all wiped surfaces achieved microbial reductions consistent with a lightly contaminated surface (≤2.5 cfu/cm^2^) [21]. This supports the method's use in evaluating manual disinfection efficacy for Phase 3 settings, where surface contamination may be minimal [9,14,21,22]. Finally, the use of both single and mixed inoculum reflected real-world microbial diversity on surfaces [23].

All in all, although laboratory-based, our internally validated method is more realistic than current standards (e.g. EN 16615:2015) and delivers meaningful results.

Routine Phase 3 testing is recommended to ensure practical relevance of disinfection protocols. While it may not yet have regulatory relevance, to do so might assess the significance and applicability of Phase 2, whose results must remain relevant for the practical use of disinfection products. Failure to do so would mean that healthcare facilities may need to validate efficacy independently, increasing workload and legal risk.

### Study limitations

Method development involved the use of reference conditions such as drying or water controls and ineffective disinfectant concentrations, which were reported where appropriate. Generalizations about disinfectant efficacy or test organisms were avoided, and specific organisms and product concentrations were selected for particular validations. The use of other target organisms, such as other bacterial species, fungi or viruses, different organic loads, as well as effective concentrations of disinfectants, could be explored following the same test principle.

The surface materials used in this study, i.e. ABS, melamine, and stainless steel, are commonly used in hospital environments and represent frequently touched surfaces therein. However, they constitute by no means a full representation of all the variety of materials that could be found in clinical settings. Even though we observed no significant differences across surface types, the results of this study should be carefully extrapolated when considering materials different than those used here (porous materials, textured surfaces, etc.).

Surface material validation used a clinical isolate of *A. baumannii*, while disinfectant application was validated using *S. aureus* ATCC 6538. As touch-transfer contamination typically occurs on dry surfaces [7,23], organisms with drying resistance—potentially conferring increased disinfectant tolerance—were used for surface testing. Conversely, *S. aureus*, a resilient, common nosocomial pathogen and a standard in disinfectant efficacy testing [23], was chosen for disinfectant validation to ensure comparability across laboratories and minimize risk to participants.

For mixed inoculum experiments, MRSA and VRE were selected as representative *Enterococcus faecium, Staphylococcus aureus, Klebsiella pneumoniae, Acinetobacter baumannii, Pseudomonas aeruginosa*, and *Enterobacter* spp. (ESKAPE) pathogens [24,25], both capable of prolonged surface survival [23] and distinguishable in co-culture without competitive overgrowth. This combination was deemed suitable for methodological validation. Although *A. baumannii* could have been used in place of VRE, it too would be a valid representative of hospital-associated contamination.

The use of different isolates for different validations may limit direct comparisons across methodological components. However, alternative organisms may yield varying results and should be considered in future studies to further refine and expand this methodology.

The simulated-used test in this study involved 6 participants, who wiped an ABS surface in triplicate. The aim of the test was to show that there are differences in the efficacy when the test surfaces were wiped by different test persons, and thus, only a small number of participants (*N* = 6) was chosen. The test did not calculate the difference in speed and pressure or their impact on efficacy. For that, further tests with more test persons would be required. The minimum number of replicates that should be performed was calculated as 9 [26,27], with CI of 95%, standard deviation of 0.7295 (Figure 4) and maximum error margin of 0.5.

## CRediT authorship contribution statement

**A. Ulatowski:** Writing – original draft, Visualization, Validation, Software, Investigation, Formal analysis, Data curation, Conceptualization. **B. Knobling:** Writing – review & editing, Validation, Supervision, Conceptualization. **D.C. Mogrovejo:** Writing – review & editing, Writing – original draft, Visualization, Formal analysis. **J.K. Knobloch:** Writing – review & editing, Supervision, Resources, Methodology, Funding acquisition, Conceptualization. **F.H.H. Brill:** Writing – review & editing, Validation, Supervision, Resources, Project administration, Funding acquisition, Conceptualization.

## Ethics statement

All test participants are part of the laboratory team of Dr. Brill + Partner GmbH. A training session explaining the aim of the study as well as the procedure was carried out before their involvement in the study. A training protocol was signed by the participants. Participation remained completely voluntary and could be withdrawn at any time. No personal information, images or any other details were collected or stored for these participants. All procedures were performed in compliance with internal institutional guidelines.

## Funding sources

This work was supported by the Freie und Hansestadt Hamburg, Behörde für Arbeit, Gesundheit, Soziales, Familie und Integration [grant number Zj92961j2020jG500–02.10/10,014].

## Conflict of interest statement

All authors declare that they have no known competing financial interests or personal relationships that could have influenced the work reported in this study. F.H.H. Brill is the owner and managing director. J. Knobloch is a member of its Scientific Advisory Board. A. Ulatowski, D.C. Mogrovejo are currently employed by Dr. Brill und Partner GmbH Institut für Hygiene und Mikrobiologie. The test persons are also all currently employed by Dr. Brill und Partner GmbH Institut für Hygiene und Mikrobiologie. The commercial products used in this study are not, nor have they ever been, manufactured by Dr. Brill und Partner GmbH Institut für Hygiene und Mikrobiologie.
