# Supplementary material for: A novel method of simulated-use surface disinfection efficacy testing as Phase 3 Step 1 approach
Source: Infect Prev Pract. 2026 Jan 20;8(2):100511. doi: 10.1016/j.infpip.2026.100511 (PMC12936730; doi:10.1016/j.infpip.2026.100511)
Supplement: Multimedia component 1 [file mmc1.docx]

**Supplementary Material**

**A novel method of simulated-use surface disinfection efficacy testing as Phase 3 Step 1 approach.**

A Ulatowski^1^, B Knobling^2^, D C Mogrovejo^1^, J K Knobloch^2^, F H H Brill ^1^

**^1^** Department of Bacteriology, Dr. Brill + Partner GmbH Institute for Hygiene and Microbiology, Hamburg, Germany.

**^2^**Institute for Medical Microbiology, Virology and Hygiene, Department Infection Prevention and Control, University Medical Center Hamburg-Eppendorf, Hamburg, Germany.

**Corresponding author:** anna.ulatowski@brillhygiene.com

**Keywords:** Surface Disinfection; Phase 3 testing; simulated-use-testing, wipes, clinical isolates, touch transfer

**Part 1.- Information about the isolates used in this study**

The clinical isolates used in this study (Supplementary Table 1) represent relevant clones of MDRO associated with outbreaks all over the world.

The Vancomycin resistant *E. faecium* ST117 isolate represents a classical hospital-associated MLST type that is widely prevalent among hospital patients worldwide [1].

The methicillin resistant isolate S. aureus ST8 (sta-type t451) belongs to the globally spreading clonal complex CC8 of MRSA [2, 3] and carries in this isolate a *qac*A gene, as it was described for isolates of this clone from human and veterinary sources [4, 5].

The Carbapenem resistant *A. baumannii* ST2 isolate represents the globally dominant lineage IC2 of CRAB isolates [6, 7]

**Supplementary Table I:** Reference strains with American Type Culture Collection (ATCC) numbers and corresponding clinical isolates.

| **Reference laboratory strain** | **Clinical isolate / MLST**** | **Resistance genotypes in clinical isolates***** |
| --- | --- | --- |
| *Enterococcus hirae**  (ATCC 6057) | *E. faecium /*  ST117 | *aac*(6')-I, *van*S-B, *van*X-B, ***van*B**, *van*H-B, *van*Y-B, *mef*(H), *msr*(C), *dfr*G, *cop*B, *pbp*5_M485A, **23S_G2576T**, *gyr*A_S83I, *par*C_S80I |
| *Enterococcus faecium**  (ATCC 10541) |  |  |
| *Staphylococcus aureus*  (ATCC 6538) | *S. aureus* (MRSA) /  ST8 (spa t451) | *aph*(3')-IIIa, *bla*PC1, ***mec*A**, *fos*B, *erm*(C), *mph*(C), *msr*(A), *sat*4, *tet*(38), *tet*(K), *dfr*G, *mep*A, ***qac*A**, *lmr*S, *mer*A, *mer*T, *mer*B, *mur*A_G257D, *fol*P_E208K, *fol*P_F17L |
| *Acinetobacter baumannii**  (ATCC 19606) | *A. baumannii /*  ST2 | *ant*(3'')-IIa, *aph*(3'')-Ib, *aph*(6)-Id, *bla*OXA-66, ***bla*OXA-23**, *bla*ADC-30, *mph*(E), *msr*(E), *tet*(B), *amv*A, *ade*C, *aba*F, *nre*B, *gyr*A_S81L |

* Included in this table for comparison purposes. Not actually used in the test described in the study.

** Multilocus sequence typing

***Genes mediating resistance against broad spectrum β-lactams, linezolid or QACs were marked in bold

References

[1] Werner et al. 2020, DOI: 10.1016/j.drup.2020.100732

[2] Deurenberg and Stobberingh, 20008, DOI: 10.1016/j.meegid.2008.07.007

[3] Robinson and Enright, 2004, DOI: 1111/j.1469-0691.2004.00768.x

[4] Lee et al., 2022, DOI: 10.1016/j.jmii.2021.08.013

[5] Worthing et. al., 2018, DOI: 10.1016/j.vetmic.2018.02.004

[6] Diancourt et al., 2010, DOI: 10.1371/journal.pone.0010034

[7] Kostyanev et al., 2021, DOI: 10.1016/j.ijantimicag.2021.106345

**Part 2.- Preparation of solutions**

**Polysorbate 80:**

1 g polysorbate 80 (Carl Roth GmbH, Germany) per 1 L hard water

**Hard water:**

Hard water was prepared by adding 6 mL/L of solution A (19.84 g/L magnesium chloride (MgCl_2_) + 46.24 g/L calcium chloride (CaCl_2_) dissolved in distilled or deionized and demineralized water) to 8 mL/L of solution B (35.02 g/L sodium bicarbonate (NaHCO_3_) dissolved in distilled or deionized and demineralized water) and adjusting the final volume to 1 L. The pH was adjusted to 7.0 ± 0.2 using 1 mol/L sodium hydroxide (NaOH) or 1 mol/L hydrochloric acid (HCL). All reagents purchased from Carl Roth GmbH (Germany).

**Part 3.- Validation over time for isolates used in study**

To validate the stability of the three isolates over time, efficacy tests and drying curves were repeatedly performed over a period greater than six months.

**Efficacy tests:**

The efficacy tests were performed according to the simulated use test developed in this study. To be able to detect any possibly changes in the sensitivity against the used disinfectant A, the tests were performed with one concentration in the effective range (1.0 % - effective concentration according to manufacturer) and one concentration in the ineffective range (0.01 % - ineffective concentration according to manufacturer). Supplementary Figure S1 shows the results of the simulated use tests with the 1.5 and 7-months old isolates (time after the isolates were received in the laboratory).


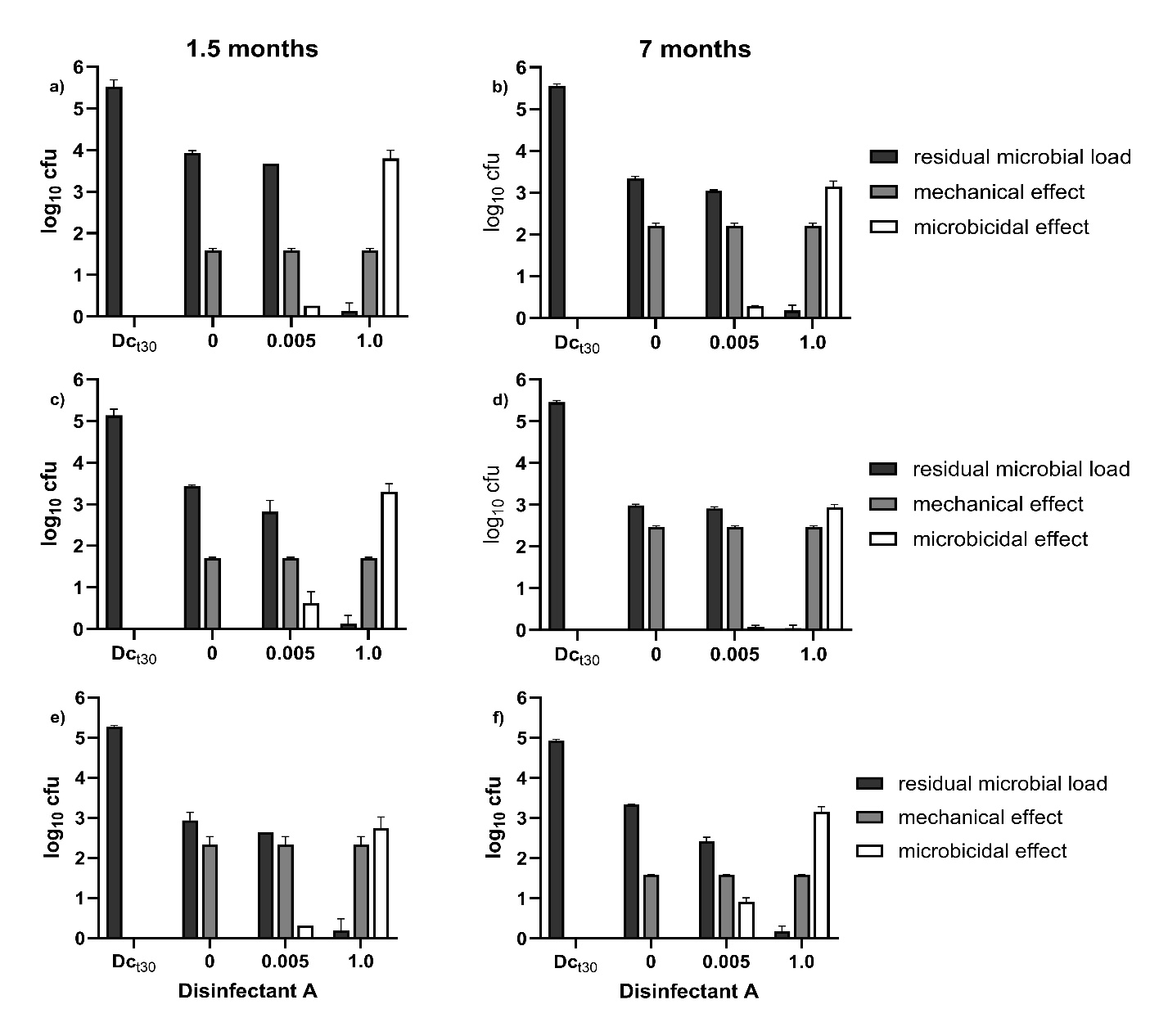


Supplementary Figure S1: Recovery of the residual microbial load (black bars) of the three clinical isolates and the mechanical (grey bars) and microbicidal effect (white bars) of disinfectant A after simulated use tests on ABS with an exposure time of 30 minutes (n=2). The tests were performed regularly over a period greater than six months. The figure shows results of the tests with 0 % (water control N_W_), 0.01% and 1.0 % disinfectant A (and the 30 minutes drying control Dc_t30_) with 1.5 and 7 months old isolates. a/b) MRSA, c/d) VRE and e/f) A. baumannii.

**Drying curves:**

The drying curves were obtained by performing drying controls with the three isolates according to the developed simulated use test over a period of approximately six months (Supplementary Figure S2). One test field of the test surface (ABS) was contaminated via touch transfer with the respective isolate and stored for drying at 20 °C (relative humidity between 40-60%) for 0, 15, 30, 45, 60 and 120 minutes. For each isolate and every drying time, two test fields were prepared and the wiping process was performed by one test person. After the corresponding drying times, the test organisms were recovered from the test fields, plated, and incubated according to EN 16615:2015.

The isolates were considered usable over the period of approximately six months when the recovery of the isolates after the respective drying times as well as after the application of disinfectant A remained within a range of 10-fold increase or decrease compared to the recovery of the first test day and at least 10^4.5^ cfu were recovered after the respective drying times.





Supplementary Figure S2: Drying curves of a) MRSA, b) VRE and c) A. baumannii 0.5 – 6.0 months after receiving the isolates in the laboratory. Shown are the recoveries of the isolates after drying according to the simulated use tests on ABS for 0 – 120 minutes at room temperature (n=2).
